# Epidemiological and clinical aspects of urogenital schistosomiasis in women, in Burkina Faso, West Africa

**DOI:** 10.1186/s40249-016-0174-1

**Published:** 2016-09-01

**Authors:** Adama Zida, Janika Briegel, Ibrahim Kabré, Marcel P. Sawadogo, Ibrahim Sangaré, Sanata Bamba, Abdourahamane Yacouba, Amado Ouédraogo, Dieudonné Yonli, François Drabo, Lady Kady Traoré, Ramata Ouédraogo-Traoré, Robert Tinga Guiguemdé, Jürgen Wacker

**Affiliations:** 1 Service de Parasitologie-Mycologie, Centre Hospitalier Universitaire Yalgado Ouédraogo, 03 BP 7022, Ouaga, 03 Burkina Faso; 2 Unité de Formation et de Recherche en Sciences de la Santé, Université de Ouagadougou, Ouagadougou, Burkina Faso; 3 Department of Obstetrics and Gynecology Bruchsal, Heidelberg University Hospital, Heidelberg, Germany; 4 Institut Supérieur des Sciences de la Santé, Université Polytechnique de Bobo-Dioulasso, Bobo-Dioulasso, Burkina Faso; 5 Ministère de la Santé, Direction de la Lutte contre la Maladie, Ouagadougou, Burkina Faso; 6 Ecole Privée de Santé Sciences Nouvelles/Centre Médical des Sciences Nouvelles, Ouagadougou, Burkina Faso; 7 Centre Médical avec Antenne chirurgical de Kombissiri, Kombissiri, Burkina Faso

**Keywords:** Urogenital schistosomiasis, Epidemiology, Clinic, Women, Burkina Faso

## Abstract

**Background:**

Because infections with *Schistosoma Haematobium* usually peak in childhood, the majority of studies on schistosomiasis have focused on school-aged children. This study aimed to assess the epidemiological and clinical aspects of urogenital schistosomiasis in women in Burkina Faso, West Africa.

**Methods:**

A cross-sectional study was conducted in a mesoendemic region (Kombissiri) and a hyperendemic region (Dori) for schistosomiasis in Burkina Faso. A total of 287 females aged 5 to 50 years were included in the study. *S. haematobium* infection was assessed using the urine filtration method and dipsticks were used for the detection of hematuria. Interviews were conducted to identify clinical aspects and risk factors related to urogenital schistosomiasis.

**Results:**

The overall prevalence of *S. haematobium* infection in Dori was 21.3 %, where as Kombissiri was less affected with a prevalence of 4.6 %. The most affected age group was the 10- to 14-year-olds (41.2 %), followed by the 15- to 19-year-olds (26.3 %). Risk factors significantly associated with schistosomiasis (*P* <0.05) were place of residence, age, contact with open water in the past year, and distance of home to open water. The percentage of participants who had contact with open water was significantly higher among the women living in Dori compared to Kombissiri. Females over 15 years of age showed a significant higher rate of water contact compared to the 5- to 15-year-olds. A significant correlation between schistosomiasis and hematuria was established. Microhematuria showed a sensitivity of 80.6 %, a specificity of 92.7 %, and a positive predictive value of 61.7 %, whereas macrohematuria had a sensitivity of 47.2 %, a specificity of 99.2 %, and a positive predictive value of 89.5 %. The mass distribution of praziquantel in Burkina Faso is well established. However, over half of the participants with schistosomiasis in this study said they took praziquantel in the past 6 months, which indicates a high reinfection rate. This may be associated with a lack of knowledge about the transmission of schistosomiasis. Only 6 % of the participants in Kombissiri and 1.5 % in Dori knew about the correct mode of transmission.

**Conclusions:**

The results of our study indicate that distribution campaigns should be extended from school-aged children to young women. Our data also demonstrate the necessity of combining already established mass distribution campaigns with information campaigns, so that long-term elimination, or at least reduction, of schistosomiasis can be achieved.

**Electronic supplementary material:**

The online version of this article (doi:10.1186/s40249-016-0174-1) contains supplementary material, which is available to authorized users.

## Multilingual abstracts

Please see Additional file 1 for translations of the abstract into the five official working languages of the United Nations.

## Background

Human schistosomiasis, a parasitic and often chronic illness, is one of the major neglected tropical diseases worldwide. It is estimated that 240 million people suffer from schistosomiasis, with more than 200000 fatalities recorded each year [1, 2]. Schistosomiasis is caused by an infection of the blood fluke *Schistosoma* and is transmitted to humans through direct contact with infected water [2].

Two forms are endemic in Sub-Saharan Africa: intestinal schistosomiasis caused by *Schistosoma mansoni*, and urogenital schistosomiasis caused by *S. haematobium*; the latter affects 112 million people [3]. The main symptoms of urogenital schistosomiasis are hematuria and dysuria due to a chronic inflammation of the bladder and urethra. Inflammation is caused by schistosome eggs that are laid into the vesical venous plexus where the adult schistosomes live. The eggs then migrate through the tissue into the bladder causing an immunological reaction [4, 5]. Complications are anemia, chronic cystitis, cancer of the bladder, and genital lesions [6, 7].

Since infections with *S. haematobium* usually peak in childhood [8], the majority of studies on schistosomiasis have focused on school-aged children. This is also the case in Burkina Faso, where an annual campaign against schistosomiasis has been conducted since 2004, part of which praziquantel is distributed, primarily to school-aged children [9].

A study on urogenital schistosomiasis in Tanzanian women showed that women’s daily contact with potentially infected water was very high, mainly due to such activities as bathing, chores, and agriculture [10]. The correlation between water contact and infection rates indicates that women are also at a high risk of contracting a schistosome infection [2, 11].

Therefore, in contrast to most studies that have been conducted in Burkina Faso, in our study we focus on women and girls, which allows us to assess the different risk factors and morbidities for females in different age groups. We compared school-aged children (5- to 15-year-olds) and adolescents and adults (over 15 years of age) to evaluate who should be target age group for the current mass distribution campaign. The sites chosen were located in one moderately endemic and one hyperendemic district in Burkina Faso, giving us the opportunity to also compare differences at a regional level.

## Methods

### Study sites

A cross-sectional study was conducted in Kombissiri in November–December 2012 and inDori in January–February 2013.

Kombissiri is the capital of the Bazèga Province, located in the central south region of Burkina Faso, and part of the Sudano-Sahelian area. The district has a hot semi-arid climate, which closely resembles a tropical wet and dry climate. The city has a rainfall of about 800 mm per year. The rainy season continues from May to October, reaching a peak from June to September. The mean average temperature is 28 °C. The cold season runs from December to January, with a minimum average temperature of 16 °C. Based on studies focusing on school-aged children, Kombissiri is considered a moderate endemic area for schistosomiasis [12, 13]. A snail survey conducted in this area found two species that are intermediate hosts of *S. haematobium*: *Bulinus senegalensis* in small reservoirs and *B. truncatus* in dams [14].

Dori, the capital of the Séno Province, is located in the Sahel region. It is characterized by a steppe climate. On average, evaporation exceeds precipitation. The average temperature is higher than 18 °C all year round. Dori has been identified as a high endemic area for schistosomiasis [12, 13]. *B. senegalensis* and *B. truncatus* are the two snail species present in this area [14].

**Fig. 1:**
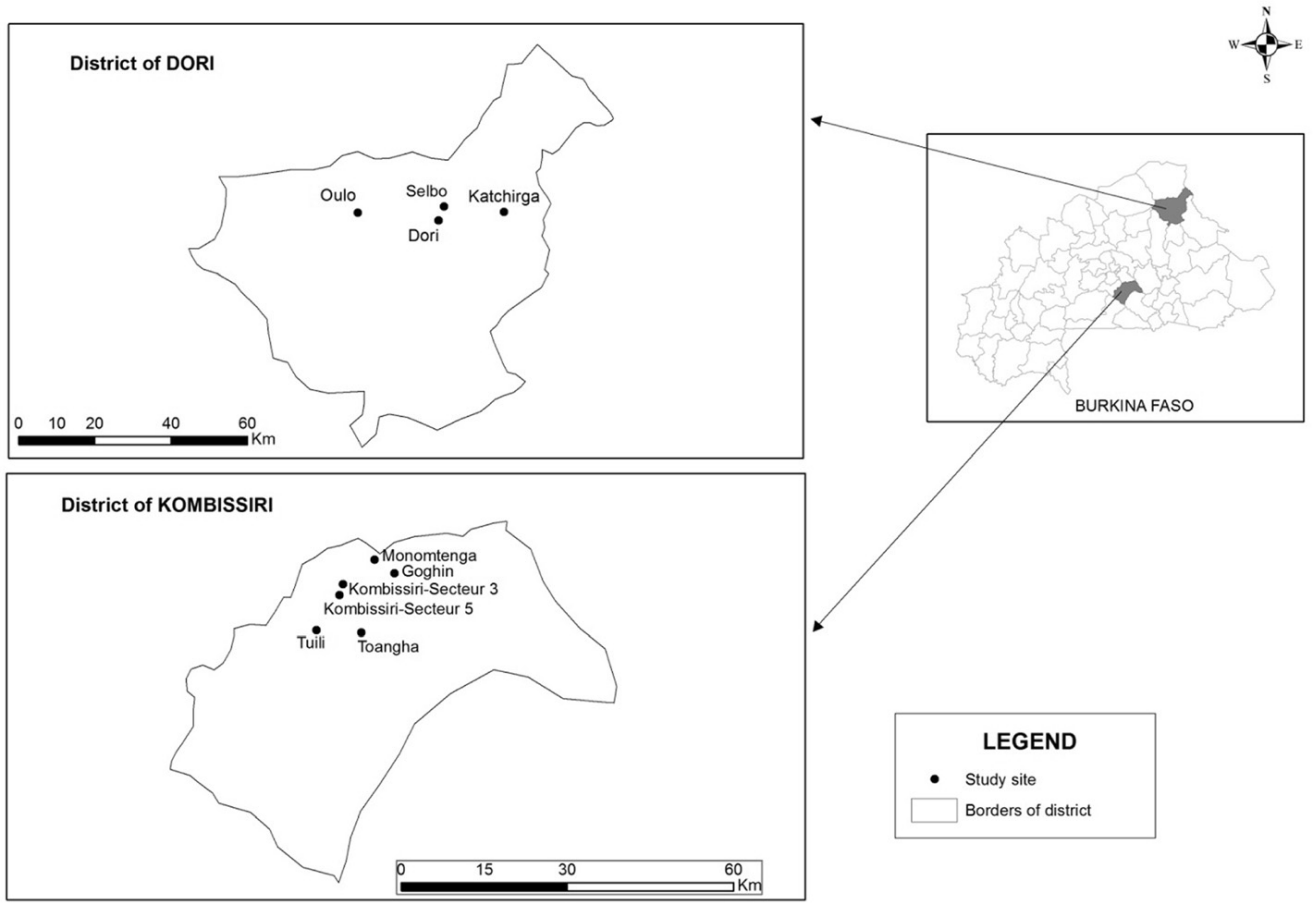
Map of the study areas and selected sites

Six study sites were chosen in Kombissiri (Monomtenga, Goanghin, Toangha, Tuili, Secteur 5, and Secteur 3) and four in Dori (Selbo, Oulo, Katchirga, and Dori Center) (see Fig. 1). These are all located near small dams.

### Sample size and sampling

A cross-sectional study was conducted with 151 women in Kombissiri and 136 women in Dori, giving a total sample size of 287 women.

With a prevalence of urogenital schistosomiasis of 10 % (a prevalence that was previously found among women in Ghana [15]), and a confidence interval of 95 % and margin of error of no more than 5 %, the desired sample size was calculated as 138 for each area. Hypothesizing a 20 % attrition rate, the final sample size of 166 was determined.

We first counted the number of concessions in each locality. We then divided the number of concessions by 166 to obtain a sampling fraction. The sampling fraction was then used to select 166 concessions among those counted in the locality. In each selected concession, a list of school-aged and adolescent girls as well as women was drawn up, and one of them was randomly selected for the study.

Females between the ages of five and 50 were selected with the help of local health workers, and gathered at the nearest health center where urine samples were taken and interviews to elucidate socio-epidemiological factors were conducted. The samples were brought to an urban laboratory, where the urine was examined for *S. haematobium* and hematuria. In case of positive findings of *Schistosoma*, the patient was treated with praziquantel (40 mg/kg) free of charge.

### Interviews

Each participant was interviewed to elucidate socioepidemiological factors related to schistosomiasis and schistosomiasis-related symptoms. The interviews were conducted by a medical student of the University of Ouagadougou in the local language and by staff members from the local health center. The questionnaire included questions on the patient’s medical history, as well as risk factors and symptoms related to schistosomiasis.

### Urine samples

A urine sample was collected from each participant between 10 am and 2 pm, and labeled with an identification number. All samples were transported to the laboratory in a cooling box. There, 10 ml of urine was filtered through a nylon filter and examined microscopically for schistosome eggs by biomedical technicians who had been trained in the national schistosomiasis control program. The detection of any *S. haematobium* eggs was considered positive, and the intensity was noted as eggs per 10 ml of urine. An intensity of 1–49 eggs/10 ml urine was graded as a light infection and ≥50 eggs/10 ml urine as a severe infection.

Whether the participant had macrohematuria (reddish coloring of urine) or microhematuria (blood in urine detected using dipstick tests) was ascertained. Microhematuria was classified using reagent strips for urine analysis (Siemens HEMASTIX®, England, London). In the case of a positive reaction, the participant was questioned about her menstrual status. If hematuria was discovered without detection of *Schistosoma* eggs, the urine of the participant was re-examined.

### Statistical analysis

Data analysis was carried out using IBM SPSS Statistics for Windows, Version 19.0. (IBM Corp., USA) to determine the frequency of the infection, various risk factors, and clinical symptoms.

These data were then analyzed using the chi-squared test. *P*-values <0.05 were considered statistically significant. If the chi-squared test could not be applied, the Fisher’s exact test was conducted.

The sensitivity and specificity of macro- and microhematuria as signs of schistosome infection were evaluated using a fourfold table.

## Results

### Prevalence

Schistosome eggs were detected in seven of the 151 participants in Kombissiri, resulting in an overall prevalence of *S. haematobium* infection of 4.6 %. Depending on the village, the prevalence ranged from 0 % (in Monomtenga, Goanghin, and Secteur 3) to 14.8 % (in Secteur 5) (see Table 1). All infections were classified as light infections, with 3–12 eggs/10 ml urine.

**Table 1:** Prevalence of urogenital schistosomiasis among women in Kombissiri and Dori (*n* = 287) in 2013

Region	Village (no. of participants)	Prevalence (pos. cases)
Kombissiri	Total (151)	4.6 % (7)
	Monomtenga (15)	0 % (0)
	Goanghin (17)	0 % (0)
	Toangha (20)	5.0 % (1)
	Secteur 5 (27)	14.8 % (4)
	Tuili (25)	8.0 % (2)
	Secteur 3 (47)	0 % (0)
Dori	Total (136)	21.3 % (29)
	Selbo (40)	42.5 % (17)
	Oulo (27)	7.4 % (2)
	Katchirga (36)	13.9 % (5)
	Dori Centre (33)	15.2 % (5)

In the villages of Dori, 29 of the 136 participants were positive for schistosomiasis, with a total prevalence of *S. haematobium* infection of 21.3 %. This ranged from 7.4 % in Oulo to 42.5 % in Selbo (see Table 1). Of those infected in Dori, 27.6 % was severely infected with over 50 eggs/10 ml urine.

**Fig. 2:**
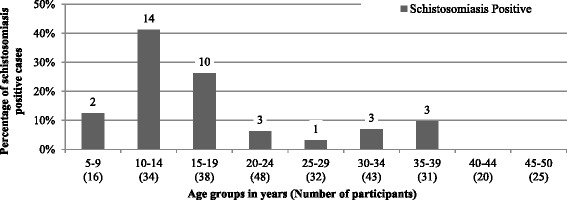
Prevalence of urogenital schistosomiasis in different age groups (*n* = 287), 2013

The group of 10- to 14-year-olds was the most affected, with 41.2 % of positive cases. A high infection rate was also observed in young women aged between 15 and 19 years: schistosome eggs were discovered in the urine of 26.3 % of this age group (see Fig. 2). None of the females over the age of 40 were found to be infected.

### Risk factors

Risk factors significantly associated with schistosomiasis infection were place of residence, young age, contact with open water during the past year, and proximity (<500 m) to an open water site.

Participants living in Dori were significantly more likely to be infected than those living in Kombissiri (see Table 1) (chi-squared test = 18.164, df = 1, *P* <0.0005). The different prevalence rates in the villages of Dori were also analyzed using the chi-squared test, revealing a significant association between the village of residence and schistosomiasis infection (chi-squared test = 15.477, df = 3, *P* = 0.001). Due to the lack of data, this test could not be performed for the villages of Kombissiri.

Children and young women aged under 20 years were significantly more likely to be infected than those aged 20 years and above (see Fig. 2) (chi-squared test = 33.44, df = 1, *P* <0.0005).

**Fig. 3:**
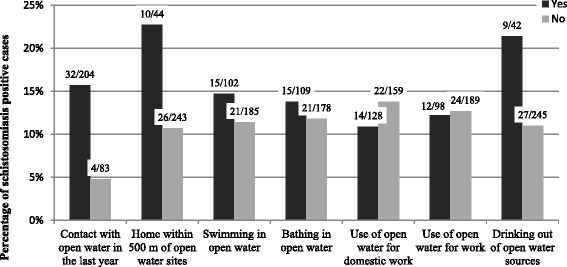
Percentage of schistosomiasis positive cases and associated risk behaviors (*n* = 287), 2013

Out of the 204 participants who stated that they had come into contact with open water in the past year, 15.7 % were infected with *S. haematobium.* In contrast, only 4.8 % were infected among the 83 participants who said that they had not come into contact with an open water source during the past year (see Fig. 3).

The chi-squared test showed a significant correlation (chi-squared test = 6.35, df = 1, *P* = 0.012) between contact with open water during the past year and the likelihood of a schistosomiasis infection.

Another risk factor found to be significantly associated with schistosomiasis was living near an open water source. Out of the 44 participants who said their home was within 500 m of an open water site, 22.7 % (10) were found to be positive for schistosomiasis.

In contrast, out of the 243 participants who stated that their home was further than 500 m away from an open water site, there were only 10.7 % (26) of positive cases (chi-squared test = 4.913, df = 1, *P* = 0.027).

No significant correlation could be determined between the frequency of infection and contact with open water sites due to swimming, bathing, using water for domestic duties/work, or drinking (*P* >0.05).

In Dori, practices related to open water were more frequent than in Kombissiri. More females in Dori stated that they had regular contact with open water due to swimming, bathing, drinking, and domestic duties, such as laundry, cooking, and cleaning, or for their work, such as fishing or agriculture. Nearly all of the participants in Dori had been in contact with water during the past year (94.9 %), whereas in Kombissiri only half of the participants (49.7 %) were. More participants in Dori lived within 500 m of an open water source than in Kombissiri.

In Dori, 37 participants (27.2 %) stated that their main source of water was water from open water sources e.g. dam or lake. In Kombissiri, only seven participants (4.6 %) participants had homes within 500 m of an open water source (see Table 2).

**Table 2:** Comparison of water contact practices among participants in Kombissiri and Dori (*n* = 287) in 2013

Water contact practices	Kombissiri (151)	Dori (136)	Chi-squared	df	*P*-value
Contact in the past year	75 (49.7 %)	129 (94.9 %)	71.07	1	<0.0005
Home within 500 m of open water	7 (4.6 %)	37 (27.2 %)	28.081	1	<0.0005
Swimming	14 (9.3 %)	88 (64.7 %)	95.981	1	<0.0005
Bathing	16 (10.6 %)	93 (68.4 %)	101.438	1	<0.0005
Domestic duties	40 (26.5 %)	88 (64.7 %)	42.294	1	<0.0005
Work	14 (9.3 %)	84 (61.8 %)	87.683	1	<0.0005
Drinking	5 (3.3 %)	37 (27.2 %)	32.703	1	<0.0005

We asked the participants what their nearest water source was and if they had access to alternative water sources e.g. wells, taps, or pumps. In total, 92 % stated that they had an alternative water source that was closer to their home than an open water source e.g. lake, dam, etc. Significantly more participants in Kombissiri (147) said they had closer alternatives, compared to those living in Dori (117) (97.4 % in Kombissiri and 86.0 % in Dori: chi-squared test = 12.442, df = 1, *P* <0.0005).

A significant association could be observed between contact with open water (swimming, bathing, domestic duties, work, drinking) and the lack of access to alternative water sources (see Table 3).

**Table 3:** Water contact practices among participants with closer access to alternative water sources compared to participants without this alternative (*n* = 287) in 2013

Water contact practices	Nocloser alternatives (23)	Closer alternatives (264)	Chi-squared	df	*p*-value
Swimming	16 (69.6 %)	86 (32.6 %)	12,636	1	<0.0005
Bathing	18 (78.3 %)	91 (34.5 %)	17,224	1	<0.0005
Domestic duties	16 (69.6 %)	112 (42.4 %)	6,308	1	0.012
Work	17 (73.9 %)	81 (30.7 %)	17,584	1	<0.0005
Drinking	14 (60.9 %)	28 (10.6 %)			<0.0005^a^

^a^ Fischer’s exact test

Those over the age of 15 more frequently stated that they had regular contact with open water due to swimming, bathing, domestic duties, their work, and drinking than school-aged children (see Table 4). This difference was significant in every aspect except for swimming. However, in general, a slightly higher percentage of school-aged children stated they had water contact with an open water source in the past year compared to adolescents and adults.

**Table 4:** Comparison of water contact practices among 5- to 15-year-olds and over 15-year-olds (*n* = 287) in 2013

Water contact practices	5–15 years (58)	>15 years (229)	Chi-squared	df	*p*-value
Contact in the past year	45 (77.6 %)	159 (69.4 %)	1.5	1	0.2207
Swimming	17 (29.3 %)	85 (37.1 %)	1.231	1	0.267
Bathing	8 (13.8 %)	101 (44.1 %)	18.052	1	<0.0005
Domestic duties	17 (29.3 %)	111 (48.5 %)	6.877	1	0.009
Work	7 (12.1 %)	91 (39.7 %)	15.756	1	<0.0005
Drinking	3 (5.2 %)	39 (17.0 %)	5.209	1	0.022

### Clinical aspects of schistosomiasis

Data collected during the interviews showed no significant associations between infection with schistosomiasis and clinical symptoms such as hematuria, dysuria, dysmenorrhea, dyspareunia, genital lesions or genital pruritus. The urine analysis, however, showed a significant association between hematuria and the presence of schistosome eggs in urine (see Tables 5).

**Table 5:** Macrohematuria and microhematuria as indications for schistosomiasis infection (*n* = 287) in 2013

	Schisto. pos. urine	Schisto. neg. urine	Total
Macrohematuria	17	2	19
No Macrohematuria	19	249	268
Total	36	251	287
Microhematuria	29	18	47
No Microhematuria	7	227	234
Total	36	245^a^	281

^a^ Six females with microhematuria did not appear for re-examination and were excluded

The Fisher’s exact test showed a significant association between schistosomiasis and macrohematuria (*P* <0.0005). Macrohematuria was visible in 17 of the 36 infected participants, thus resulting in a sensitivity of 47.2 %. Out of the 251 females without schistosome eggs in the urine, 249 were also negative for macrohematuria, resulting in a specificity of 99.2 %. The positive predictive value for macrohematuria was 89.5 %.

The chi-squared test revealed a significant association between schistosome eggs in the urine and blood detected using dipstick tests (chi-squared test = 120.888, df = 1, *P* <0.0005). Microhematuria was detected in 29 of the 36 infected participants, thus resulting in a sensitivity of 80.6 %. Out of the 245 examined females without schistosome eggs, 227 were also negative for microhematuria, resulting in a specificity of 92.7 %. The positive predictive value for microhematuria was 61.7 %.

### Mass distribution campaign and praziquantel intake

In total, 33.5 % of the participants stated that they had taken praziquantel during the past 6 months (32.9 % in Kombissiri and 34.1 % in Dori).

**Fig. 4:**
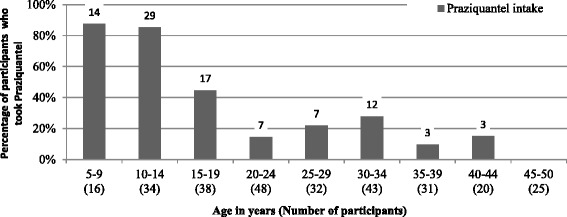
Age-dependent intake of praziquantel during the past 6 months (*n* = 287), 2013

The majority of school-aged participants stated that they had taken praziquantel during the past 6 months (see Fig. 4). The 5- to 9-year-olds were the group with the highest intake (87.5 %), followed by the 10- to 14-year-olds (85.3 %). Almost half (44.7 %) of the 15- to 19-year-olds had taken praziquantel. The older age groups all had an intake of less than 30 %.

Out of the participants with schistosome eggs in the urine, 55.6 % (20/36) stated that they had taken praziquantel during the past 6 months. Out of the participants without a detectable infection, 28.7 % (72/251) said that they had taken praziquantel.

### Knowledge of schistosomiasis

In total, 75.6 % of the participants said they had heard of schistosomiasis (93.4 % in Dori and 59.6 % in Kombissiri). When the participants were asked how one can become infected with schistosomiasis, only 3.8 % knew about the correct transmission mode: contact with open water sources (1.5 % in Dori and 6.0 % in Kombissiri). No significant differences could be observed between the different age groups and knowledge of schistosomiasis.

## Discussion

Many previous studies on schistosomiasis in Burkina Faso have been summarized by Poda et al. [12], and all showed prevalence rates of schistosomiasis between 0 and 100 %. These different prevalence rates indicate a high heterogeneity of the distribution of the disease.

The present study also showed significant differences between the two examined regions: with an infection rate of 21.3 %, Dori was more strongly affected than Kombissiri (4.6 %). The significant differences between villages confirm previous observations of strong local variations even within endemic areas [16, 17].

The lower prevalence in this study in comparison to some previous studies could indicate the effectiveness of the mass distribution campaigns, which have been conducted since 2004 [9].

However, when comparing the results of this study with previous studies, it should be noted that the age profile of the current study (20.2 % are 5- to 15-year-olds) differs from most previous studies performed in Burkina Faso, which have focused on school-aged boys and girls [9, 18–20]. According to Institut National de la Statistique et de la Démographie (National Institute of Statistics and Demography) 48 % of the population in Burkina Faso is under 15 years of age [21].

When looking at the prevalence rates among the different age groups, it becomes clear that children and young women are especially affected (see Fig. 1). In addition to be more often infected children had more contact with open water sources than adolescents and adults (77.6 % vs. 69.4 %) (see Table 4). These results are consistent with findings of several authors who stated that the higher prevalence for schistosomiasis in children is often associated with more frequent water contact [22, 23]. However, in this study the rate of adolescents and adults who had more contact with open water sources remains high (69.4 %). Many other authors explain this fact by an acquired immunity [24–28].

One should also consider that the sensitivity of microscopic egg detection depends on many factors e.g. intensity of infection, the time the sample was collected, and the number of examined samples [29, 30]. As demonstrated by Poggensee et al. [31], many cases of urogenital schistosomiasis stay undetected when the examination method is limited to urine filtration. They discovered *Schistosoma* eggs in 23 % of the cases where the urine filtration method showed a negative result. Hegertun et al. [32] made similar observations.

There are many possible explanations for the different prevalence rates in Dori and Kombissiri. A part from age and region, the main factors associated with schistosomiasis infection include different population of intermediate hosts in the open water sites and behaviors of inhabitants relating to contact with open water [22, 26, 33, 34]. The inhabitants of Dori used open water sources much more frequently than those living in Kombissiri. Nearly all the inhabitants in Dori had been in contact with an open water source during the past year, whereas in Kombissiri less than half were. The significant association was observed between last contact with an open water source and infection with schistosomiasis, indicating that this may be an important factor in the difference in infection rates in Kombissiri and Dori.

The type of water contact has also been acknowledged as an important risk factor. The amount of the body surface exposed, the duration of exposure, and type of water source all influence the risk of infection [22, 35]. An increased risk due to activities where large amounts of body surface are exposed has been described in a number of publications [24, 36–38].

This study showed no significant increased risk of infection with respect to the different kinds of water contact practices. However, a higher prevalence could be observed in those who drank the water. A possible explanation is that those who are willing to drink water from open water sources also probably have a higher tendency to engage in other risky behaviors.

There was no increase of risk among participants who regularly used an open water source for domestic duties or their work. This has also been described by other authors [37, 38]. A possible explanation for this was given by Rudge et al. [37], who postulated that the use of soap or detergent kills cercaria. Another reason could be that skin exposure while engaged in domestic duties is low, which thus keeps the risk of infection low [24].

In terms of the different age groups, the 5– to 15-year-olds had more frequent water contact than the older subjects. This is consistent with the majority of studies that state that children are at a higher risk of infection due to more frequent water contact [22, 39]. A study performed by Chandiwana [24], however, showed more frequent water contact among those aged over 15 years in comparison to children.

A significant correlation between the lack of alternative water sources and use of open water sources was observed. This has also been described in other studies that demonstrated that the presence of alternative water sources lead to a reduction of contact with potentially infectious water and thus decreased the infection rate [40–42].

The fact that a home is near an open water source has also been discussed as an important risk factor for infection [16, 37, 43]. Rudge et al. [37] postulated that the distance from an infectious water source is more important to assess the risk of infection than the given information about contact with open water. The results of our study also show a significant association between the distance of a home from an open water source and risk of schistosomiasis infection.

Clinical symptoms related to schistosomiasis include genital symptoms e.g. genital lesions, pruritus, dyspareunia and dysmenorrhea [7, 10, 44]; and urinary symptoms e.g. dysuria and hematuria [8, 45–47].

There are many possible explanations for the lack of associations between schistosomiasis and clinical symptoms in the present study. As sexual infectious diseases with similar clinical symptoms are not uncommon in Burkina Faso [48], misinterpretations can easily occur. Even though female genital mutilation has been forbidden in Burkina Faso, most of the interviewed women stated that they were circumcised, which can cause confusion in terms of genital symptoms. An infection with schistosomiasis without eggs detected in the urine could also lead to a misinterpretation [31]. Another factor is the possibility of the underreporting of symptoms. This has also been described by other authors, especially in relation to older girls and hematuria [49, 50]. A possible explanation is the onset of menstruation [50]. One must also consider that these questions are very intimate and it is possible that many of the participants preferred to not admit to any kind of urogenital symptoms.

A significant association could, however, be established between detected hematuria and schistosomiasis infection. This is a common association and has even been postulated as a reliable diagnostic method [15, 51–53].

The high positive predictive value for macro- and microhematuria underline the statement of their use in the field as these methods are very cost- and time effective.

Since 2004, national mass distribution campaigns with praziquantel have been implemented, focusing on school-aged children [9]. This has been confirmed in this study, in which the majority of school-aged children stated that they had received and taken praziquantel during the past 6 months. The fact that those with schistosomiasis infection more frequently stated that they had taken praziquantel during the past 6 months than those without infection indicates that the distribution of the drugs is focusing on the appropriate group. However, the fact that over half of those with a detectable schistosomiasis infection had taken praziquantel beforehand could also be due to other factors: either these patients had been infected and the parasites had not been fully eliminated, they are now (re)infected, or the statement that they gave relating to taking praziquantel was incorrect. A 2-year follow-up study conducted by Touré et al. [19] showed a significant decline of infection after praziquantel intake, demonstrating the effectiveness of a one-dose medication. It is, however, known that praziquantel has no effect in juvenile stages [54–56], which explains why a second dose of praziquantel after 3 to 6 weeks has been recommended by Garba et al. [55]. A possible resistance has also been discussed [54].

The lack of knowledge about the transmission mode and exposure to possibly infected water sites could indicate the possibility of a high reinfection rate. Considering that campaigns against schistosomiasis have been underway since 2004 and campaign posters have been visible in many of the rural health centers, the lack of knowledge is striking. A lack of knowledge about water-associated diseases in the population of Burkina Faso has also been noted by Boelee et al. [34]. Asaolu and Ofoezie [40] also emphasize that health education is essential for a long-term reduction of infection rates.

To further reduce the infection rate, contact with potentially infectious water sites should be limited and further contamination of open water sites should be prevented. This can be done by installing sanitary facilities [40]. A report by the United Nations International Children’s Emergency Fund and the World Health Organization [57] showed that in the rural areas of Burkina Faso, 76 % of the inhabitants did not have access to sanitary facilities and 76 % defecated openly.

## Conclusion

Even though the prevalence reported in this study was lower than reported in many previous studies, it can be said that urogenital schistosomiasis is still a serious health problem in certain regions of Burkina Faso. Furthermore, this study indicates that adolescents and adults are also frequently infected, highlighting that the focus of mass distribution campaigns should be reconsidered and possibly extended beyond school-aged children.

Due to an undefined number of undetected cases, studies using different methods are necessary to provide further information on the extent of the infection. It is also advised that additional studies are conducted to investigate and control reinfection rates.

To prevent new cases and reinfection, and to achieve a long-term reduction or even elimination of schistosomiasis, public awareness must be raised. This could be integrated into the already existing mass distribution campaigns.

## Additional file

Additional file 1: Multilingual abstract in the five official working languages of the United Nations. (PDF 435 kb)
