# Supplementary material for: Epidemiological and clinical aspects of urogenital schistosomiasis in women, in Burkina Faso, West Africa
Source: Infect Dis Poverty. 2016 Sep 1;5(1):81. doi: 10.1186/s40249-016-0174-1 (PMC5007686; doi:10.1186/s40249-016-0174-1)
Supplement: Additional file 1: — Multilingual abstract in the five official working languages of the United Nations. (PDF 435 kb) [file 40249_2016_174_MOESM1_ESM.pdf]

Translation of the abstract into the five official working languages of the United Nations

الجوانب الوبائية والسريرية لمرض البلهارسيا البولية التناسلية لدى النساء، في بوركينا فاسو، غرب أفريقيا

-اسماء اشخاص-: اداما زيدا، جانيكا بريجل، ابراهيم كابري، مارسيل ب ساوادوجو، ابراهيم سانجاري، ساناتا بامبا، ادوراهماني ياكوبا، امادو اودراجو، ديودوني يونلي، فرانسوا درابو، ليدي كيدي تروري، راماتا اودراجو-تروري، روبرت تينجا جيجيمدي، يورجين واكر

ملخص

خلفية: لأن العدوى مع البلهارسيا البولية عادة ما ذروتها في مرحلة الطفولة، فإن غالبية الدراسات حول مرض البلهارسيا قد ركزت على الأطفال في سن المدرسة. هدفت هذه الدراسة إلى تقييم الجوانب الوبائية والسريرية لمرض البلهارسيا البولية التناسلية لدى النساء في بوركينا فاسو، غرب أفريقيا

الطرق: أجريت دراسة مستعرضة في منطقة خطرة مرضيا في **(Kombissiri)** والمنطقة بشكل مفرط (دوري) لمرض البلهارسيا في بوركينا فاسو. أدرجت ما مجموعه 287 الإناث الذين تتراوح أعمارهم بين 5-50 سنوات في الدراسة. وجرى تقييم العدوى الدموية باستخدام طريقة الترشيح البول واستخدمت الغميسة للكشف عن بيلة دموية. وأجريت مقابلات لتحديد الجوانب السريرية وعوامل الخطر ذات الصلة بالبلهارسيا البولية التناسلية.

النتائج: معدل انتشار العدوى الدموية في دوري كانت 21.3٪، في حين كانت أقل تأثرا في Kombissiri مع انتشار 4.6٪. وكانت الفئة العمرية الأكثر تضررا من الذين تتراوح أعمارهم بين 14 سنة-10- إلى (41.2٪)، يليها الفئة العمرية 19 عاما 15- إلى (26.3٪ (P <0.05) .(وكانت عوامل الخطر المرتبطة بشكل كبير مع البلهارسيا في مكان الإقامة والعمر، والاتصال مع المياه المفتوحة في العام الماضي، والمسافة من المنزل إلى فتح المياه. وكانت نسبة المشاركين الذين كانوا على اتصال مع المياه المفتوحة أعلى بكثير بين النساء اللاتي يعشن في دوري مقارنة في .Kombissiri وأظهرت الإناث أكثر من 15 عاما من العمر على أعلى معدل كبير من ملامسة الماء مقارنة مع الذين تتراوح أعمارهم بين 15 عاما 5- ل. وتم إنشاء ارتباط كبير بين البلهارسيا وبيلة دموية. أظهرت بيلة دموية مجهرية حساسية من 80.6٪، وخصوصية 92.7٪، والقيمة التنبؤية الإيجابية من 61.7٪، في حين كان بيلة دموية مجهرية حساسية من 47.2٪، وخصوصية 99.2٪، والقيمة التنبؤية الإيجابية من 89.5٪. راسخة توزيع كتلة برازيكوانتل في بوركينا فاسو. ومع ذلك، قال أكثر من نصف المشاركين بمرض البلهارسيا في هذه الدراسة أخذوا برازيكوانتل في الأشهر الستة الماضية، وهو ما يشير إلى معدل الإصابة مرة أخرى عالية. قد تترافق هذه مع نقص في المعرفة حول انتقال مرض البلهارسيا. 6٪ فقط من المشاركين في Kombissiri و 1.5٪ في دوري عرف عن الوضع الصحيح للنقل.

الاستنتاجات: إن نتائج دراستنا تشير إلى أن حملات التوزيع يجب أن تمتد من الأطفال في سن المدرسة للشابات. كما تظهر البيانات المتوفرة لدينا على ضرورة الجمع بين حملات توزيع كتلة القائمة بالفعل مع الحملات الإعلامية، بحيث القضاء على المدى الطويل، أو تخفيض على الأقل، من البلهارسيا يمكن أن يتحقق.

Translated from English version into Arabic by Bamo A. Aziz, through

TRANSLATORS
WITHOUT BORDERS

西非布基纳法索女性尿路血吸虫病的流行病学和临床表现

Adama Zida, Janika Briegel, Ibrahim Kabré, Marcel P Sawadogo, Ibrahim Sangaré, Sanata Bamba, Abdourahamane Yacouba, Amado Ouédraogo, Dieudonné Yonli, François Drabo, Lady Kady Traoré, Ramata Ouédraogo-Traoré, Robert Tinga Guiguemdé, Jürgen Wacker

摘要：

引言：因为埃及血吸虫感染经常发生在儿童时期，所以大部分的研究集中于学龄儿童。本研究的目的是评估西非布基纳法索女性尿路血吸虫病的流行病学和临床表现。

方法：本研究在布基纳法索的一个中度流行区（孔比西里）和一个高度流行区（多里）进行横断面研究。

共有 287 名年龄在 5-50 岁的女性被纳入研究。采用尿液过滤法和试纸条法检测血尿和埃及血吸虫感染情况。此外，还通过采访受试者来确认临床表现和尿路血吸虫病的相关危险因素。
结果：多里地区总的尿路血吸虫病发病率为 21.3%，而孔比西里的发病率仅为 4.6%。受影响最严重的年龄组为 10-14 岁（41.2%），其次为 15-19 岁（26.3%）。居住地、年龄、在过去几年与开放水源接触等风险因素与尿路血吸虫病显著相关（*P*<0.05）。多里地区接触开放水源的 5-15 岁女性受试者明显高于孔比西里地区，15 岁以上的受试者与水源的接触率明显高于 5-15 岁受试者群体。血吸虫感染与血尿症显著性相关。镜下血尿症检测特异性为 92.7%，敏感性为 80.6%，阳性预测值为 61.7%，而肉眼血尿症检测特异性为 47.2%，敏感性为 99.2%，阳性预测值为 89.5%。吡喹酮在布基纳法索各地已经建立了完整的大规模分发渠道，本研究超过半数的血吸虫病患者陈述他们在过去的半年内曾服用过吡喹酮，这表明复发率很高。这或许与缺乏血吸虫病传播相关知识有关。孔比西里、多里分别仅有 6%和 1.5%的受试者知晓正确的血吸虫病传播模式。
结论：研究结果表明，药物分配活动应该从学龄儿童扩大到年轻女性。我们的数据表明已有的大规模分发活动和信息活动相结合的必要性，只有在此基础之上才能实现血吸虫病的长期消除，或者至少是下降。

Translated from English version into Chinese by Feng Xinyu, edited by Yang Pin, through

CHINESE CENTER FOR DISEASE CONTROL AND PREVENTION
NATIONAL INSTITUTE OF PARASITIC DISEASES

Aspects épidémiologiques et cliniques de la schistosomiase urogénitale chez la femme au Burkina Faso, en Afrique de l'Ouest

Adama Zida, Janika Briegel, Ibrahim Kabré, Marcel P Sawadogo, Ibrahim Sangaré, Sanata Bamba, Abdourahamane Yacouba, Amado Ouédraogo, Dieudonné Yonli, François Drabo, Lady Kady Traoré, Ramata Ouédraogo-Traoré, Robert Tinga Guiguemdé, Jürgen Wacker

Résumé
Contexte : Comme les infections par *Schistosoma haematobium* sont habituellement plus nombreuses pendant l'enfance, la majorité des études sur la schistosomiase se sont concentrées sur les enfants d'âge scolaire. La présente étude explore les aspects épidémiologiques et cliniques de la schistosomiase urogénitale chez la femme au Burkina Faso (Afrique de l'Ouest).
Méthodes : Une étude transversale de la schistosomiase a été réalisée dans une région mésoendémique (Kombissiri) et une région hyperendémique (Dori) au Burkina Faso. Au total, 287 femmes âgées de 5 à 50 ans ont été incluses dans l'étude. Les infections par *S. haematobium* ont été estimées en utilisant la méthode de filtration des urines et des bandelettes réactives ont été utilisées pour détecter l'hématurie. Des entretiens ont été menés pour déterminer les aspects cliniques et les facteurs de risque de la schistosomiase urogénitale.
Résultats : La prévalence totale des infections par *S. haematobium* dans la région de Dori était de 21,3 %, tandis que Kombissiri était moins affectée avec une prévalence de 4,6 %. La classe d'âge la plus affectée était les 10-14 ans (41,2 %), suivies par les 15-19 ans (26,3 %). Les facteurs de risque associés de façon significative à la schistosomiase ($P < 0,05$) étaient le lieu de résidence, l'âge, le contact avec de l'eau libre au cours de l'année passée et la distance du domicile à un point d'eau libre. Le pourcentage de participantes en contact avec de l'eau libre était significativement plus élevé dans la région de Dori qu'à Kombissiri. Les femmes âgées de 15 ans montraient un taux de contact avec l'eau libre significativement plus élevé que les filles de 5 à 15 ans. Une corrélation significative a pu être établie entre la schistosomiase et l'hématurie. La microhématurie a montré une sensibilité de 80,6 %, une

spécificité de 92,7 % et une valeur prédictive positive de 61,7 %, tandis que la macrohématurie avait une sensibilité de 47,2 %, une spécificité de 99,2 % et une valeur prédictive positive de 89,5 %. La distribution en masse de praziquantel au Burkina Faso est bien établie, mais plus de la moitié des participantes à l'étude porteuses d'une schistosomiase ont dit avoir pris du praziquantel dans les six derniers mois, ce qui indique un fort taux de réinfection peut-être lié à un manque de connaissances sur la transmission de la schistosomiase. Seules 6 % des participantes de Kombissiri et 1,5 % de Dori connaissaient le mode de transmission.

Conclusions : Les résultats de notre étude indiquent que les campagnes de distribution devraient être étendues des enfants d'âge scolaire aux jeunes femmes. Nos données montrent également la nécessité de doubler les campagnes de distribution de masse déjà en place de campagnes d'information si l'on veut éliminer, ou du moins réduire, la schistosomiase à long terme.

Translated from English version into French by Suzanne Assenat, through

TRANSLATORS
WITHOUT BORDERS

Эпидемиологические и клинические аспекты мочеполового бильгарциоза среди женщин в Буркина Фасо, Западная Африка.

Адама Зида, Яника Бригель, Ибрахим Кабре, Марсель П. Сауадого, Ибрахим Сангаре, Саната Бамба, Абдурахаман Якуба, Амадо Оуедраого, Дьюдонн Йонли, Франсуа Драбо, Лэди Кади Траоре, Рамата Оуедраого-Траоре, Роберт Тинга Гуигуемде, Юрген Уакер

Резюме

Предпосылки: Учитывая то, что заражение мочеполовым шистосомозом обычно происходит в детском возрасте, большинство исследований бильгарциоза проводилось среди детей школьного возраста. Данное исследование нацелено на изучение эпидемиологических и клинических аспектов мочеполового бильгарциоза у женщин в Буркина Фасо, Западная Африка.

Методы: Статическое исследование бильгарциоза проводилось в мезоэндемичном регионе (Комбиссири) и гиперэндемичном регионе (Дори) в Буркина Фасо. В исследовании приняли участие 287 женщин в возрасте от 5 до 50 лет. Для определения наличия инфекции использовался метод клубочковой фильтрации с использованием тест-полосок для определения крови в моче. Для определения клинических аспектов и факторов риска мочеполового бильгарциоза проводились опросы.

Результаты: Распространенность шистосомоза в Дори составила 21.3%, в то время как в Комбиссири меньше - 4.6%. Наиболее подверженными заражению оказались девочки в возрасте 10 - 14 лет (41.2%), и 15 - 19 лет (26.3%). Факторами риска, непосредственно связанными с бильгарциозом (<0.05), являются место жительства, возраст, факт наличия контакта с открытой водой за прошедший год, расстояние от дома до открытой воды. Процент участников, имевших контакт с открытой водой, выше среди жительниц Дори, нежели Комбиссири. У женщин старше 15 лет контакт с водой был чаще, чем у девочек 5-15 лет. Выявлена выраженная корреляция между бильгарциозом и гематурией. Чувствительность анализа на микрогематурию составила 80.6%, специфичность критерия - 92.7%, прогностичность положительного результата - 61.7%, чувствительность анализа на макрогематурию составила 47.2%, специфичность критерия - 99.2% и

прогностичность положительного результата - 89.5%. В Буркина Фасо широко налажен сбыт празиквантела. Однако, более половины участников данного исследования, страдающих бильгарциозом, принимали празиквантел в последние полгода, что указывает на высокий уровень реинфицирования. Это может быть вызвано недостаточной осведомленностью о путях передачи бильгарциоза. Только 6% участников из Комбиссири и 1.5% из Дори знали о путях передачи.

Выводы: Результаты исследования указывают на необходимость внедрения системы распределения как среди школьниц, так и среди молодых женщин. Кроме того, полученные данные указывают на необходимость совмещать имеющиеся системы распределения с информационными кампаниями, что позволит добиться долгосрочного избавления или как минимум сокращения случаев бильгарциоза.

Translated from English version into Russian by Ms Zhdanova, through

Aspectos epidemiológicos y clínicos sobre la esquistosomiasis urogenital en las mujeres de Burkina Faso (África Occidental)

Adama Zida, Janika Briegel, Ibrahim Kabré, Marcel P Sawadogo, Ibrahim Sangaré, Sanata Bamba, Abdourahamane Yacouba, Amado Ouédraogo, Dieudonné Yonli, François Drabo, Lady Kady Traoré, Ramata Ouédraogo-Traoré, Robert Tinga Guiguemdé, Jürgen Wacker

Resumen

Información de referencia: Debido a que las infecciones por *Schistosoma Haematobium* suelen alcanzar su máximo nivel durante la infancia, los estudios sobre esquistosomiasis se centran en su mayoría en niñas en edad escolar. Este estudio tenía como objetivo evaluar los aspectos epidemiológicos y clínicos sobre la esquistosomiasis urogenital en las mujeres de Burkina Faso (África Occidental).

Métodos: Se realizó un estudio de carácter transversal en una región mesoendémica (Kombissiri) y en una región hiperendémica (Dori) de la esquistosomiasis en Burkina Faso. En el estudio participaron 287 hembras con edades entre 5 y 50 años. Se evaluó la infección por *S. haematobium*, utilizando para ello el método de filtración de orina y varillas para la detección de hematuria. Se llevaron a cabo entrevistas para identificar los aspectos clínicos y factores riesgo relacionados con la esquistosomiasis urogenital.

Resultados: La prevalencia general de la infección por *S. haematobium* en Dori fue del 21,3%, mientras que Kombissiri estaba menos afectada, con una prevalencia del 4,6%. El grupo de edad más afectado fue el de 10 a 14 años (41,2%), seguido por el de 15 a 19 años (26,3%). Los factores de riesgo relacionados significativamente con la esquistosomiasis (P<0,05) fueron el lugar de residencia, edad, contacto con aguas abiertas durante el pasado año, y distancia entre el hogar y aguas abiertas. El porcentaje de participantes en contacto con aguas abiertas fue significativamente más alto entre las mujeres que vivían en Dori respecto a las de Kombissiri. Las hembras de más de 15 años de edad mostraron una tasa significativamente más alta del contacto con agua si se compara con las de 5 a 15 años. Se estableció una correlación significativa entre la esquistosomiasis y la hematuria. La microhematuria mostró una sensibilidad del 80,6%, una especificidad del 92,7% y un valor predictivo positivo del 61,7%, mientras que la macrohematuria tenía una sensibilidad del 47,2%, una especificidad del 99,2% y un valor predictivo positivo

del 89,5%. La distribución de praziquantel en Burkina Faso es bien conocida. No obstante, más de la mitad de los participantes con esquistosomiasis en este estudio declaró haber contraído praziquantel durante los seis últimos meses, lo cual indica una elevada tasa de reinfección. Esto podría estar relacionado con la falta de conocimientos acerca de la transmisión de la esquistosomiasis. Solo el 6% de los participantes en Kombissiri y el 1,5% en Dori conocían el modo correcto de transmisión.

Conclusiones: Los resultados de nuestro estudio indicaron que las campañas de distribución deberían extenderse de las niñas en edad escolar a mujeres jóvenes. Nuestros datos también demuestran la necesidad de combinar campañas de distribución ya consolidadas y campañas de información, por lo que se puede lograr la eliminación a largo plazo o al menos la reducción de la esquistosomiasis.

Translated from English version into Spanish by SergioLorenzi, through

TRANSLATORS
WITHOUT BORDERS
